# Identification and genomic characterization of *Pseudomonas* spp. displaying biocontrol activity against *Sclerotinia sclerotiorum* in lettuce

**DOI:** 10.3389/fmicb.2024.1304682

**Published:** 2024-03-07

**Authors:** Daphné Albert, Antoine Zboralski, Marie Ciotola, Mélanie Cadieux, Adrien Biessy, Jochen Blom, Carole Beaulieu, Martin Filion

**Affiliations:** ^1^Saint-Jean-sur-Richelieu Research and Development Centre, Agriculture and Agri-Food Canada, Saint-Jean-sur-Richelieu, QC, Canada; ^2^Department of Biology, Faculty of Science, Université de Sherbrooke, Sherbrooke, QC, Canada; ^3^Bioinformatics and Systems Biology, Justus-Liebig-Universität Giessen, Giessen, Germany

**Keywords:** *Pseudomonas*, *Sclerotinia*, biocontrol, antibiosis, mycin, peptin, brabantamide, whole-genome sequencing

## Abstract

Lettuce is an economically major leafy vegetable that is affected by numerous diseases. One of the most devastating diseases of lettuce is white mold caused by *Sclerotinia sclerotiorum*. Control methods for this fungus are limited due to the development of genetic resistance to commonly used fungicides, the large number of hosts and the long-term survival of sclerotia in soil. To elaborate a new and more sustainable approach to contain this pathogen, 1,210 *Pseudomonas* strains previously isolated from agricultural soils in Canada were screened for their antagonistic activity against *S. sclerotiorum*. Nine *Pseudomonas* strains showed strong *in vitro* inhibition in dual-culture confrontational assays. Whole genome sequencing of these strains revealed their affiliation with four phylogenomic subgroups within the *Pseudomonas fluorescens* group, namely *Pseudomonas corrugata*, *Pseudomonas asplenii*, *Pseudomonas mandelii*, and *Pseudomonas protegens*. The antagonistic strains harbor several genes and gene clusters involved in the production of secondary metabolites, including mycin-type and peptin-type lipopeptides, and antibiotics such as brabantamide, which may be involved in the inhibitory activity observed against *S. sclerotiorum*. Three strains also demonstrated significant *in planta* biocontrol abilities against the pathogen when either inoculated on lettuce leaves or in the growing substrate of lettuce plants grown in pots. They however did not impact *S. sclerotiorum* populations in the rhizosphere, suggesting that they protect lettuce plants by altering the fitness and the virulence of the pathogen rather than by directly impeding its growth. These results mark a step forward in the development of biocontrol products against *S. sclerotiorum*.

## 1 Introduction

Lettuce (*Lactuca sativa* L.) is one of the most economically important leafy vegetable worldwide and an important source of dietary antioxidants (Kim et al., 2016). In Canada, lettuce is in the top 10 field grown vegetables, bringing in about US$80 million worth of harvested products, and is in the top 5 exported vegetables (Crops Horticulture Division, 2023). Québec is the largest producing province in Canada, growing 90% of the country’s lettuce (Statistics Canada, 2022). However, several diseases and infections limit the cultivation of this vegetable (Subbarao et al., 2017). One of the most economically damaging ones is white mold, caused by the necrotrophic, ubiquitous phytopathogenic fungus *Sclerotinia sclerotiorum* (Lib.) de Bary (Bolton et al., 2006; Kamal et al., 2016; Subbarao et al., 2017).

*Sclerotinia sclerotiorum* is a polyphagous fungus belonging to the *Ascomycota* family that can infect more than 400 host plants, mainly dicotyledons, as well as some monocotyledons (Bolton et al., 2006; Derbyshire et al., 2017). Aside from lettuce, this pathogen affects many economically important crops, such as canola/oilseed rape and soybean (Bolton et al., 2006; Derbyshire et al., 2017). The fungus can infect plants at any growth stage, including seedlings and mature plants, which makes it very destructive in the field (O’Sullivan et al., 2021). Infection often starts with the formation of an appressorium, which allows the fungus to penetrate through the plant cuticle (Hegedus and Rimmer, 2005). This appressorium originates either from hyphae in the soil or from the germination of an ascospore on plant aboveground tissues (Smolińska and Kowalska, 2018). *S. sclerotiorum* kills host cells by secreting cell wall degrading enzymes and toxins, such as oxalic acid, causing various symptoms in plants (Subbarao et al., 2017; Wang et al., 2019). Infected leaves usually display water-soaked lesions that rapidly spread to the stem (Bolton et al., 2006). The lesions develop typically into a white mycelium, which is the tell-tale symptom of this fungal infection. The survival structures of the fungus, sclerotia, are formed in/on the infected tissues (Bolton et al., 2006). They are the primary resting stage of the fungus, representing over 90% of its life cycle (O’Sullivan et al., 2021). When the soil conditions are not favorable for germination, the sclerotia may remain viable for at least 5 years (Smolińska and Kowalska, 2018).

The large number of hosts and the long-term survival of sclerotia in soil limit the control methods against white mold. The use of synthetic fungicides is the primary approach to control the pathogen (Smolińska and Kowalska, 2018). However, the available fungicides can be harmful to the environment, and application coverage and timing are rarely optimal, reducing efficacy (Wang et al., 2015; Hou et al., 2018; O’Sullivan et al., 2021). More importantly, due to the single mode of action and overuse of most fungicides, resistance development in *S. sclerotiorum* populations is now common (O’Sullivan et al., 2021).

The difficulty to control this fungus and limit economic losses has driven research into alternative control methods, such as biocontrol (Panpatte et al., 2016; O’Sullivan et al., 2021). Several studies have investigated potential biocontrol agents against *S. sclerotiorum*, as recently reviewed by our research team (Albert et al., 2022). Various mechanisms used by biocontrol agents have demonstrated some effectiveness against *S. sclerotiorum*. Among them, one of the most promising is antibiosis, which refers to the inhibition of pathogens through the release of antimicrobial compounds (Arseneault and Filion, 2017), such as lipopeptides, hydrogen cyanide (HCN), bacteriocins, and antibiotics. These compounds can affect a fungus by interrupting DNA replication, altering the electron transport chain, disrupting energy production, and inhibiting membrane function (Dimkić et al., 2022). Most studies performed to date on antibiosis against *S. sclerotiorum* have been conducted under *in vitro* conditions. Only few studies have demonstrated some biocontrol activity *in planta*. *Pseudomonas brassicacearum* DF41 and *Pseudomonas chlororaphis* PA23 have been shown to suppress *S. sclerotiorum*-mediated stem rot in canola, probably through the biosynthesis of lipopeptides, pyrrolnitrin, and HCN (Berry et al., 2010; Selin et al., 2010). *P. chlororaphis* PA23 also displays antifungal activity against *S. sclerotiorum* in lettuce, producing HCN to affect the pathogen (Nandi et al., 2017).

Currently, Pseudomonads, and more specifically strains belonging to the *Pseudomonas fluorescens* group, seem to be the main biocontrol agents studied against *S. sclerotiorum* in *in planta* assays involving antibiosis (Albert et al., 2022). Further research is however still needed before *Pseudomonas* spp. can be reliably used in the field against white mold in lettuce. *Pseudomonas* spp. display a multitude of traits that contribute to their biocontrol potential, including their ability to live in various habitats, such as the phyllosphere and the rhizosphere, and to produce a myriad of antifungal metabolites (Panpatte et al., 2016). However, the production of such antifungal metabolites *in situ* is known to be partly impacted by environmental conditions (D’aes et al., 2010). Therefore, isolating potential biocontrol *Pseudomonas* strains from ecosystems close to those in which they will be applied is an approach that is more likely to succeed. Not only these organisms are adapted to these environmental conditions, but their inoculation will also most likely not cause major perturbations in the native microbial community structure.

In this context, this study intended to (i) characterize the antagonistic abilities of soil-dwelling *Pseudomonas* strains against *S. sclerotiorum*, (ii) determine their ability to control the development of white mold *in planta*, and (iii) use whole-genome sequencing and comparative analyses to identity potential genetic determinants involved in their biocontrol capabilities.

## 2 Materials and methods

### 2.1 *In vitro* dual-culture confrontation tests

A collection of 1,210 *Pseudomonas* strains was assessed for their *in vitro* antagonistic abilities against *S. sclerotiorum*. These strains were previously isolated from agricultural soils in the province of Québec, Canada, and screened against three lettuce bacterial pathogens (Zboralski et al., 2022). A single pathogenic strain of *S. sclerotiorum* called Ss2019-1 was used in this study. It was isolated from a lettuce plant (cultivar Sunbelt) at L’Acadie Experimental Farm (Agriculture and Agri-Food Canada, Saint-Jean-sur-Richelieu, QC, Canada) using standard isolation procedures.

Petri plates containing potato dextrose agar (PDA) (BD Biosciences) medium were inoculated with 5-mm agar discs of 3-day-old *S. sclerotiorum* mycelium. *Pseudomonas* strains were grown in King’s B broth for 24–36 h at 24°C with agitation at 150 RPM.

The *S. sclerotiorum* 5-mm agar discs were placed in the middle of PDA plates and two drops (20 μl each) of *Pseudomonas* spp. cultures adjusted to 1 × 10^8^ CFU ml^–1^ were placed at an equal distance of 30 mm from the mycelial discs, whereas no drops were added to the control Petri plates. All Petri plates were kept at 22.5°C in the dark until the mycelium reached the edges of the control plates. Inhibition zones were then measured for all treatments. The *in vitro* inhibition abilities of each isolated *Pseudomonas* strains were tested in triplicates.

### 2.2 Genomic DNA extraction

The *Pseudomonas* strains displaying the strongest inhibitory activity against *S. sclerotiorum* were grown on King’s B agar plates incubated for 48 h at 22.5°C for genomic extraction. In parallel they were also streaked on King’s B agar plates and grown for 7 days at 25°C to assess their fluorescence at 365 nm using a UV light (Cole Parmer Instruments, Vernon Hills, IL, USA). Genomic DNA was extracted using cells harvested from the King’s B agar plate with the DNeasy UltraClean Microbial Kit (Qiagen, Mississauga, ON, Canada) following the manufacturer’s instructions with an additional step: a vortex homogenization step was preceded by an initial homogenization using the MOBIO PowerLyzer™ 24 Bench Top Bead-Based Homogenizer (VWR, Radnor, PA, USA) at a speed of 3,400 RPM for 45 s. DNA concentration was measured using a Qubit fluorometer (Invitrogen, Burlington, ON, Canada).

### 2.3 Genome sequencing, assembly, and annotation

Genomic DNA libraries were prepared using PacBio SMRTbell Express Template Prep kit (Pacific Biosciences, Menlo Park, CA, USA) with the *Pseudomonas* strains displaying the strongest inhibitory activity against *S. sclerotiorum*. Genome sequencing was performed at the Integrated Microbiome Resource (Halifax, NS, Canada) using a PacBio Sequel sequencer (v3 chemistry) as described in Biessy et al. (2022). The genomes were assembled using the Flye v2.8.1 long-read assembler (Kolmogorov et al., 2019). Default parameters were used for assembly. Annotation of the genomes was performed using the NCBI Prokaryotic Genome Annotation Pipeline v5.3 (Tatusova et al., 2016).

### 2.4 Species-level identification and phylogeny

Species-level identification of the strains that demonstrated strong inhibition against *S. sclerotiorum* was performed using the Type (Strain) Genome Server (Meier-Kolthoff and Göker, 2019; Meier-Kolthoff et al., 2022), which provides digital DNA–DNA hybridization (dDDH) values between a query genome and a set of closely related type strain genomes. Genome BLAST Distance Phylogeny (GBDP) formula d_4_ (Meier-Kolthoff et al., 2013) was used to calculate the digital DDH values. A phylogenomic tree was built out of 2,079 shared genes (for a total of 2,033,174 bp per genome) using the phylogenomic analysis performed by the EDGAR 3.0 web-server (Blom et al., 2009; Dieckmann et al., 2021). The alignment of the orthologous genes found in all genomes was performed using MUSCLE (Edgar, 2004) and the resulting alignments were concatenated into a single one. A phylogenomic tree was then generated using this alignment and the neighbor-joining method implemented in PHYLIP (Felsenstein, 2005).

### 2.5 Identification of genes and clusters responsible for secondary metabolite production potentially involved in biocontrol

Nucleotide sequences of genes and biosynthetic gene clusters (BGCs) of interest were retrieved from the *Pseudomonas* genome database (Winsor et al., 2016) and from GenBank. They were used as baits to identify numerous genes and/or BGCs in the genomes of the inhibitory *Pseudomonas* strains under study. Identification of additional secondary metabolites BGCs was performed using antiSMASH v6.0 (Blin et al., 2021). The different mycin and peptin lipopeptide variants potentially produced by the strains under study were identified using antiSMASH peptide sequence prediction. Identification of antibacterial proteins by a proteome-wide analysis of Pfam domains was conducted using the built-in Pfam domain search (v1.2) in CLC Genomics Workbench v21.0.5 (Qiagen, Aarhus, Denmark) and the Pfam-A v35.0 database (Mistry et al., 2021). Proteins harboring Pfam domains of interest were retrieved and investigated with InterProScan v2.0 (Quevillon et al., 2005) in Geneious Prime 2022.1.1 (Biomatters, Auckland, New Zealand).

For a more in-depth comparative analysis, the results of this gene identification process were compared with those obtained from 35 other *Pseudomonas* spp. in a previous study (Zboralski et al., 2022; listed in Supplementary Table 1). These strains belong to the same collection of 1,210 strains but are non-antagonistic against *S. sclerotiorum*. This comparative analysis was performed to identify potential genetic determinants underlying *S. sclerotiorum* antagonism.

### 2.6 Assessment of potential plant pathogenic effects of *Sclerotinia*-inhibiting *Pseudomonas* strains

Lettuce seeds (cultivar Sunbelt) were sown in Pro-mix BX (Premier Tech, Rivière-du-Loup, QC, Canada) and placed in a germinator under an 18/6 h day/night photoperiod with a light intensity of 300 μmol.m^–2^.s^–1^, at 22/18°C and 60% constant relative humidity. Seven days following sowing, the substrate of the lettuce seedlings, as well as lettuce leaves, were each inoculated with 10 ml of each *Sclerotinia*-inhibiting *Pseudomonas* strain grown as previously described (1 × 10^8^ CFU ml^–1^) or 10 ml of water for the control treatment. Lettuce leaves were visually inspected for the appearance of symptoms 2 weeks after inoculation to determine any adverse effects that inoculation of *Pseudomonas* spp. might have on the plants. Four replicates were used per treatment.

### 2.7 *In planta* experiment: detached leaves

Lettuce seeds were sown in the same condition as described above. After 22 days, seedling leaves of 8 cm in length were cut, washed in sterile water, and placed on absorbent paper to remove excess water. Leaves were either submerged for 1 min in each inhibitory strain of *Pseudomonas* spp. (as described above) or in sterile water (control). Each treated leaf was placed in a Petri dish lid on a sterile and humidified 90 mm Whatman filter paper (Cole-Parmer, QC, Canada). Each Petri was then sealed and placed in a multistage growth chamber (PGC20, Conviron, Winnipeg, MN, Canada) at a constant 21°C, relative humidity of 75%, light intensity of 200 μmol.m^–2^.s^–1^, and a photoperiod of 18/6 h (light/dark). Twenty-four hours later, the leaves of each bacterial treatment were inoculated with *S. sclerotiorum*. A 5-mm diameter disk of sterile Whatman filter paper was submerged in a 1-week-old culture of *S. sclerotiorum* grown in potato dextrose broth (PDB) (BD Biosciences) medium under agitation at 200 RPM at 22.5°C (ground plug suspension). Each leaf was inoculated with a disk placed near the leaf tip, between veins. Petri dishes were sealed and returned to the growth cabinet under the previously described conditions. Lesion diameters were measured 6 days after inoculation with *S*. *sclerotiorum*. Five replicates were used per treatment and the experiment was repeated a second time.

### 2.8 *In planta* experiment: pot-grown plants

*Pseudomonas* strains shown to be effective in the biocontrol of *S. sclerotiorum* in the assay on detached leaves were further selected to be tested in a pot assay. Lettuce seeds were sown using the same conditions as described above. Seven days after sowing, the growing substrate was inoculated with 10 ml of antagonistic *Pseudomonas* strains or with 10 ml of water for the control treatment. The *Pseudomonas* strains were previously grown in King’s B broth for 24–36 h at 24°C with agitation at 150 RPM (1 × 10^8^ CFU ml^–1^). Two weeks following *Pseudomonas* inoculation, plants were transplanted in 4.5-inch diameter pots filled with 150 g of growing substrate complemented with a *S. sclerotiorum* inoculum. This inoculum was prepared as follows: sterile 5-mm cracked wheat (25 g of wheat and 25 ml of distilled water) was inoculated with five 5-mm PDA discs collected from 3-day-old *S. sclerotiorum* mycelium culture. It was incubated in the dark for 72 h at 22.5°C and was shaken every 24 h. Sterile cracked wheat without *S. sclerotiorum* was also incubated for the control treatment. Inoculated wheat was then air dried for 24 h, crushed, and passed through a sieve to obtain pieces between 75 and 850 μm. The inoculum was mixed with Pro-mix BX to reach a concentration of 0.2% of the total weight of the final growing substrate for all treatments. Transplanted plants were then grown in climate-controlled chambers (PGC20, Conviron) under the same conditions as the ones used in the germinator. Eight days following transplantation, symptoms were assessed on all lettuce plants using the following five categories: (1) healthy leaves; (2) wilt leaves; (3) infected crown; (4) spreading infection; and (5) dead leaves. To assess *Sclerotinia* population levels in the rhizosphere by quantitative PCR (qPCR), 25 ml of substrate surrounding the roots (rhizosphere samples) were sampled from each pot immediately following symptom assessment. Twenty replicates were used per treatment and the experiment was repeated a second time.

### 2.9 Quantification of *Sclerotinia* population levels in the pot assay by quantitative PCR

DNA was extracted from 100 mg of each rhizosphere sample collected in the pot assay described above using Griffith’s modified DNA extraction protocol (Griffiths et al., 2008). Extraction was followed by a purification step involving the DNeasy PowerClean Pro Cleanup kit to remove any PCR inhibitors (Qiagen, Hilden, Germany). DNA quality was checked using a NanoDrop 2000 (Thermo Fisher Scientific, Waltham, MA, United States).

Quantification of *S. sclerotiorum* populations was performed by qPCR using the SSBZ primers and TaqMan probe (Ziesman et al., 2016). They specifically amplify a 70 bp-fragment within the *ssv263* gene in *S. sclerotiorum*, a hypothetical secreted protein involved in virulence (Liang et al., 2013). Their sequences are as follow: primer SSBZF, 5′-GCT CCA GCA GCC ATG GAA-3′; primer SSBZR, 5′-TGT TGA AGC AGT TGA CGA GGT AGT-3′ (Integrated DNA Technologies, Coralville, IA, United States); probe SSBZP, 5′-CAG CGC CTC AAG C-3′ (Applied Biosystems, Waltham, MA, United States). The probe harbors a 6-carboxyfluorescein (6-FAM) reporter dye at the 5′ end as well as a nonfluorescent minor groove-binding at the 3′ end (MGBNFQ). For absolute quantification, each 96-well qPCR plate contained serially diluted standards of known concentrations consisting of a purified pCR4-TOPO vector in which the 70 bp-amplicon was inserted using the TOPO TA Cloning kit (Thermo Fisher Scientific). Each qPCR reaction contained iTaq Universal Probes Supermix 1× (Bio-Rad, Hercules, CA, United States), 200 nM of both primers and the probe, and 2.4 μl of sample DNA, for a final reaction volume of 10 μl. The following thermocycles were used: an initial step at 95°C for 2 min followed by 40 two-step cycles consisting of 95°C for 5 s and 60°C for 30 s. qPCR cycling and optical reading was performed using an AriaMx Real-time PCR System (Agilent Technologies, Santa Clara, CA, United States). All samples were tested in triplicates. Each plate contained negative-control reaction wells in which the same qPCR mix was used but DNA was replaced by sterile distilled water.

### 2.10 Statistical analyses

Statistical analyses were performed using Rstudio version 1.4.1717 (Boston, MA, United States). The R function “kruskal” from the “agricolae” package version 1.3-5 involving Fisher’s least significant difference (LSD) test was used to perform multiple comparisons between treatments for the confrontation and for the detached leaves assays (de Mendiburu, 2017). The R function “gao” from the “nparcomp” package version 4.2.2 was used to perform non-parametric multiple comparisons of symptom levels between each bacterial treatment and the control in the pot assay (Gao et al., 2008). These comparison procedures were performed using the Benjamini–Hochberg *p*-value adjustment method. An ANOVA followed by a Tukey’s *post-hoc* test was used to compare the qPCR results obtained from the pot assay.

## 3 Results

### 3.1 Nine *Pseudomonas* displayed antagonistic abilities in dual-culture confrontational tests

**FIGURE 1:**
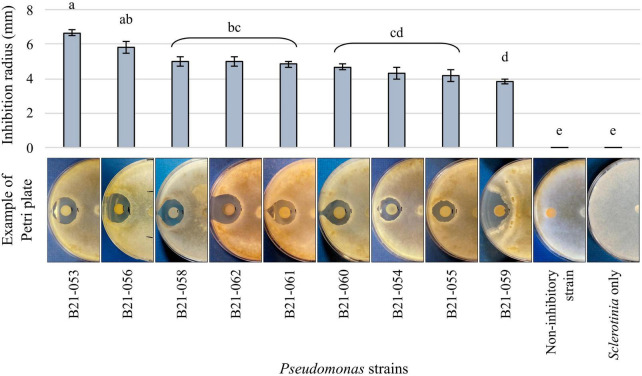
Antagonistic capabilities of nine *Pseudomonas* strains against *S. sclerotiorum in vitro*. The control plates contain the fungus alone and an example of the antagonism observed with a non-antagonistic *Pseudomonas* strain is also presented. Bars indicate standard error of the mean. Letters refer to the significantly different groups calculated using Fisher’s least significant difference procedure.

Among the 1,210 *Pseudomonas* strains tested in dual-culture confrontational tests, nine strains, representing less than 1%, displayed inhibition zones against *S. sclerotiorum* (Figure 1). The strain that displayed the highest inhibition was B21-053, with an average inhibition zone of 6.7 mm compared to strain B21-059 which exhibited the smallest average inhibition zone of 4 mm.

### 3.2 Genome sequencing revealed strains belonging to four subgroups within the *P. fluorescens* group

The genomic features of each of the nine *Pseudomonas* strains are summarized in Supplementary Table 2. The average number of reads was 601,632 and the average length of these reads was 4,310 pb. Each genome was completely assembled into one circular chromosome. Of the nine strains, only B21-055 harbors a plasmid, which is 75-kb long. Genome size ranged from 6.13 to 7.13 Mb and GC content varied from 59.5% to 62.3%.

**FIGURE 2:**
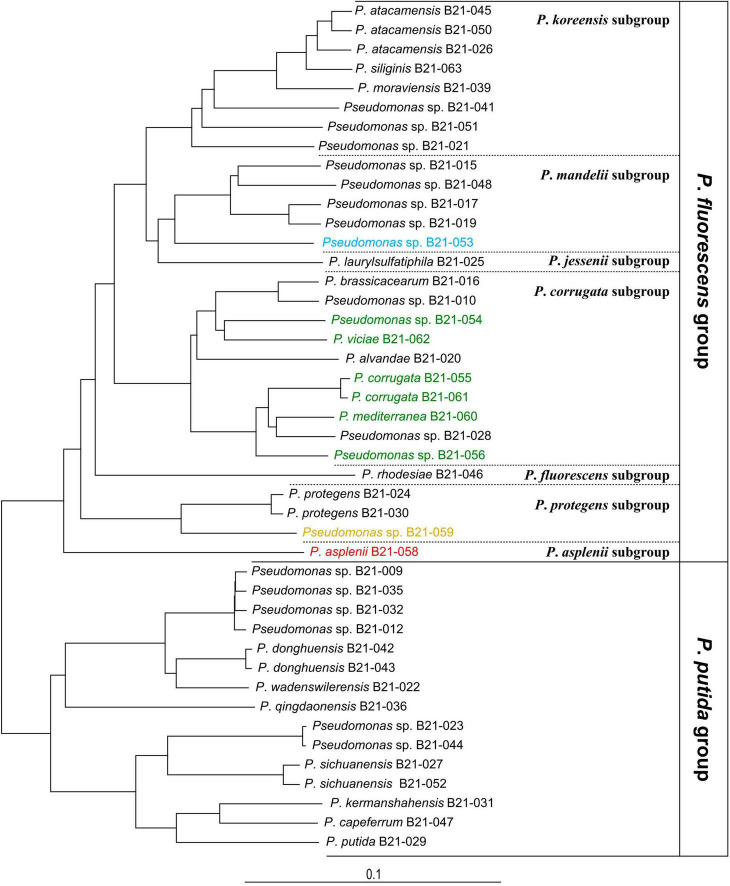
Phylogenomic tree of 44 *Pseudomona*s strains including 9 antagonistic strains (colored) and 35 non-antagonistic strains unable to inhibit *S*. *sclerotiorum*, all isolated from the same pool of soil samples. Each color refers to a *Pseudomonas* phylogenomic subgroup: green, *P. corrugata*; blue, *P. mandelii*; orange, *P. protegens*; red, *P. asplenii*. *Pseudomonas aeruginosa* DSM 50071^T^ was used as an outgroup (not shown). The bar indicates sequence divergence.

Genome sequencing of the nine *Pseudomonas* strains that displayed antagonistic activity against *S. sclerotiorum* uncovered their phylogenomic affiliation to the group, subgroup, and sometimes species levels within the *Pseudomonas* genus (Supplementary Figure 1). Figure 2 shows the phylogenomic relationships between the antagonistic strains and non-antagonistic *Pseudomonas* strains isolated from the same collection of 1,210 strains (Zboralski et al., 2022). The antagonistic strains all clustered within the *P. fluorescens* group and are subdivided into the four following subgroups: six strains belong to the *Pseudomonas corrugata*, one strain to the *Pseudomonas mandelii*, one strain to the *Pseudomonas asplenii*, and one strain to the *Pseudomonas protegens* subgroup. Five strains could be assigned to four *Pseudomonas* species, while the remaining four could not be matched to any type strain (Table 1). Each one potentially represents a new species. Four of the nine strains, all belonging to the *P. corrugata* subgroup (B21-055, B21-056, B21-060, and B21-061), did not produce fluorescence on King’s B medium.

**TABLE 1:** Taxonomic affiliation of the nine antagonistic *Pseudomonas* strains inhibiting *S. sclerotiorum* and GenBank accession numbers of their genomes.

Phylogenomic subgroup	Species name	Strain	Town of isolation in Quebec, Canada	GenBank accession number
*P. mandelii*	*Pseudomonas* sp.	B21-053	Ormstown	CP087205
*P. corrugata*	*Pseudomonas* sp.	B21-054	Ormstown	CP087204
	*P. corrugata*	B21-055	Farnham	CP102177; CP102178
	*Pseudomonas* sp.	B21-056	Farnham	CP087203
	*P. mediterranea*	B21-060	Saint-Bernard-de-Lacolle	CP102176
	*P. viciae*	B21-062	Hemmingford	CP087200
	*P. corrugata*	B21-061	Stanbridge East	CP102175
*P. asplenii*	*P. asplenii*	B21-058	Farnham	CP087202
*P. protegens*	*Pseudomonas* sp.	B21-059	Mont-Saint-Gregoire	CP087201

### 3.3 Genes and clusters of genes involved in the biosynthesis of secondary metabolites potentially inhibitory to *S. sclerotiorum* have been identified in the genomes

**FIGURE 3:**
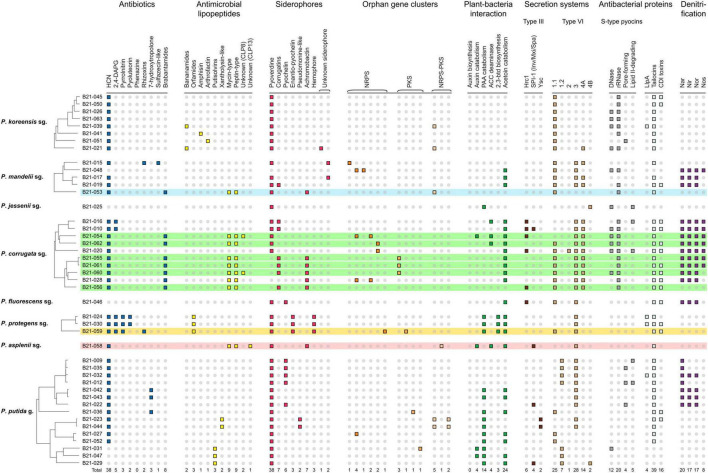
Genomic atlas of 9 *Pseudomonas* strains antagonistic to *S. sclerotiorum* (colored) and 35 non-antagonistic ones (non-colored). Each small square represents the presence of a biosynthetic gene or gene cluster involved in pathogen suppression, ecological competence and/or plant-growth promotion. HCN, hydrogen cyanide; 2,4-DAPG, 2,4-diacetylphloroglucinol; NRPS, non-ribosomal peptide synthase; PKS, polyketide synthase; PAA, phenylacetic acid; ACC, 1-aminocyclopropane-1-carboxylate; 2,3-btd, 2,3-butanediol; Llpa, lectin-like putidacin A; CDI, contact-dependent inhibition; sg., subgroup; g., group.

To better understand the mechanisms involved in the inhibition of *S. sclerotiorum* by the nine antagonistic strains, the distribution of genes and gene clusters known for their role in the production of secondary metabolites involved in biocontrol was investigated (Figure 3). This analysis included 35 other *Pseudomonas* spp. characterized in a previous study (Zboralski et al., 2022; Supplementary Table 1), which are non-antagonistic against *S. sclerotiorum*. This comparative approach aimed to identify differences in gene distribution between antagonistic and non-antagonistic strains, which could point to genetic determinants involved in *S. sclerotiorum*’s inhibition.

Interestingly, eight of the nine strains that displayed *in vitro* antagonistic activity against *S*. *sclerotiorum* harbored BGCs involved in the production of mycin and peptin antimicrobial lipopeptides, as well as the antibiotic brabantamide. These BGCs were not found in the genomes of 35 *Pseudomonas* strains unable to inhibit *S. sclerotiorum*, except for one (B21-028). The following mycin and peptin lipopeptides were identified *in silico* as potential products of these BGCs: nunamycin, brasmycin, thanamycin, syringotoxin, thanamycin, nunapeptin, braspeptin, corpeptin, and fuscopeptin (Table 2).

**TABLE 2:** Mycin and peptin lipopeptides potentially produced by antagonistic *Pseudomonas* strains.

Strain	Subgroup	Species	Mycin	Peptin
B21-053	*P. mandelii*	*Pseudomonas* sp.	Nunamycin	Nunapeptin
B21-054	*P. corrugata*	*Pseudomonas* sp.	Brasmycin/thanamycin^1^	Unknown^2^
B21-062		*P. viciae*	Brasmycin^1^	Braspeptin
B21-055		*P. corrugata*	Thanamycin^1^	Corpeptin
B21-061		*P. corrugata*	Thanamycin^1^	Corpeptin
B21-060		*P. mediterranea*	Thanamycin^1^	Corpeptin
B21-056		*Pseudomonas* sp.	Thanamycin-var^3^	Unknown^2^
B21-059	*P. protegens*	*Pseudomonas* sp.	None	None
B21-058	*P. asplenii*	*P. asplenii*	Syringotoxin	Fuscopeptin

The prediction of the various compounds was performed using antiSMASH 7.0. The predicted amino acid sequences were compared with previously characterized mycin and peptin molecules to determine which compounds the nine strains under study might produce.

^1^Brasmycin and thanamycin have the same *in silico* amino acid prediction (Girard et al., 2020), so prediction of their production was also based on phylogenetic distance and gene-for-gene identity to known producing strains. In the case of B21-054, it was impossible to accurately predict which of the two compounds was potentially produced.

^2^The predicted amino acid sequences of the compounds do not match any previously characterized molecules.

^3^The predicted compound is similar to Thanamycin-var1 produced by *Pseudomonas* sp. DF41 (Girard et al., 2020).

All inhibitory strains and almost all non-inhibitory ones used for comparison harbored the HCN cluster. The inhibitory strains also carry clusters involved in the biosynthesis of five different siderophores: pyoverdine, corrugatins, enantio-pyochelin, achromobactin, and hemophore. Each inhibitory strain carried one to three of these siderophore clusters in its genome. The BGCs encoding two types of type III secretion systems (T3SS) and three types of type VI secretion systems (T6SS) were also identified in the nine strains, along with various antibacterial proteins.

The strains belonging to the *P. protegens* subgroup were the only ones among the 44 strains under study to harbor the BGCs responsible for pyrrolnitrin, pyoluteorin, orfamides, enantio-pyochelin, hemophore, and 2,3-butanediol production. The only inhibitory strain identified in this subgroup in this study, *Pseudomonas* sp. B21-059, carried the largest number of antibiotics biosynthetic genes and BGCs among the nine antagonistic strains, encoding the enzymes involved in the biosynthesis of 2,4-diacetylphloroglucinol (2,4-DAPG), pyrrolnitrin and rhizoxin (Figure 3).

### 3.4 The nine inhibitory strains do not cause any visible symptoms on plants

The *Pseudomonas* genus encompasses various plant-pathogenic strains, including within phylogenomic groups known for their numerous biocontrol strains. To ensure that the nine antagonistic *Pseudomonas* strains under study are not pathogenic to lettuce, pathogenicity was assessed by inoculating the different strains on lettuce plants to verify the appearance/development of symptoms and/or stresses. No symptoms or stresses were observed.

### 3.5 Three *Pseudomonas* strains displayed *in planta* biocontrol activity against *S. sclerotiorum*

**FIGURE 4:**
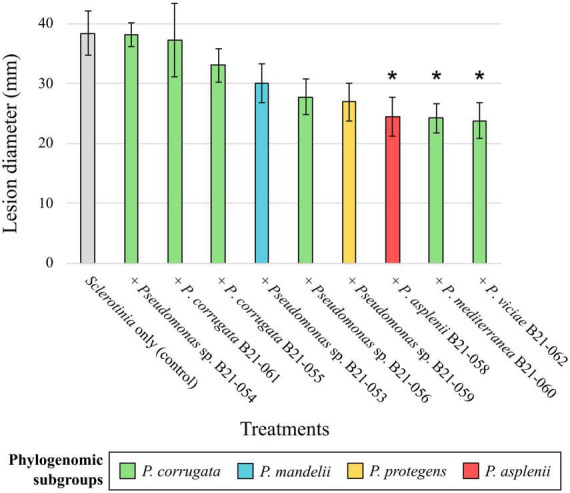
Biocontrol capabilities of nine *Pseudomonas* strains against *S. sclerotiorum* on detached leaves. The colors refer to subgroup affiliations and the control: gray, *S. sclerotiorum* (control); green, *P. corrugata*; blue, *P. mandelii*; orange, *P. protegens*; red, *P. asplenii*. Asterisk indicates a significant difference between a bacterial treatment and the control, with *p* < 0.05, resulting from Fisher’s least significant difference procedure. Bars indicate the standard error of the mean.

To assess the *in planta* inhibitory abilities of the nine *Pseudomonas* strains that displayed antagonistic activity under *in vitro* conditions, co-inoculations of each strain with *S. sclerotiorum* were performed on detached lettuce leaves. The mean lesion diameters caused by *S. sclerotiorum* co-inoculated with the nine strains under study are shown in Figure 4, varying between 23.8 and 38.2 mm. Leaves inoculated with strains B21-058, B21-060, and B21-062 displayed significantly smaller (*p* < 0.05) lesions compared to the control inoculated with *S. sclerotiorum* only, reducing lesion diameters by 36% to 38%.

**FIGURE 5:**
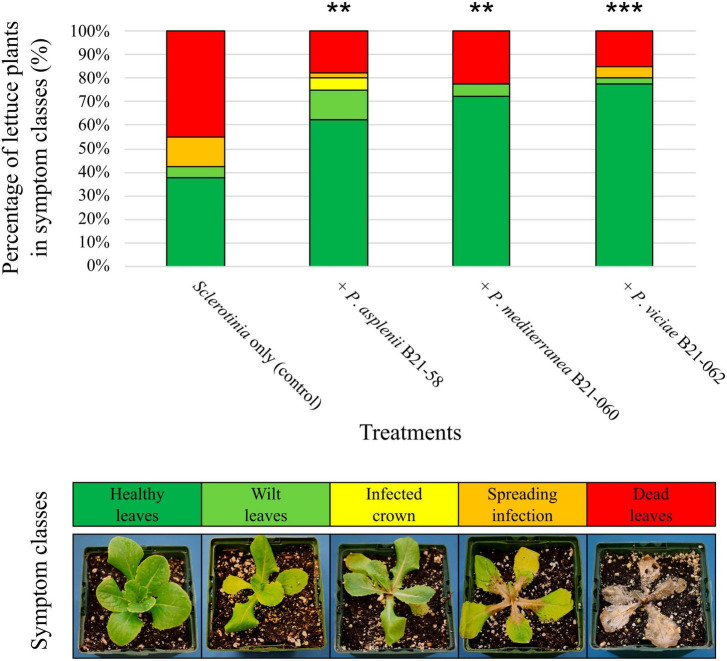
Biocontrol capabilities of three *Pseudomonas* strains against *S. sclerotiorum* in pot-grown lettuce. An example of plant in each symptom class is shown at the bottom. The symbols “**”or “***” indicate a significant difference between a bacterial treatment and the control, with *p* < 0.01 or *p* < 0.001, respectively, resulting from the non-parametric multiple comparison procedure of Gao et al. (2008).

These same three *Pseudomonas* strains were then inoculated in the growing substrate of lettuce seedlings, which were subsequently transferred into pots filled with *S. sclerotiorum*-inoculated substrate and grown in controlled chambers. Eight days after pathogen inoculation, all three strains significantly decreased symptom intensity on lettuce compared to the plants inoculated with *S. sclerotiorum* only (Figure 5). The decrease in symptom intensity ranged from 38% for B21-058 to 45% for B21-062. Between 63% and 78% of the plants inoculated with a *Pseudomonas* strain displayed healthy leaves, while only 38% of control plants did.

### 3.6 Population levels of *S. sclerotiorum* in the lettuce rhizosphere were not affected by the inoculation with *Pseudomonas* strains

**FIGURE 6:**
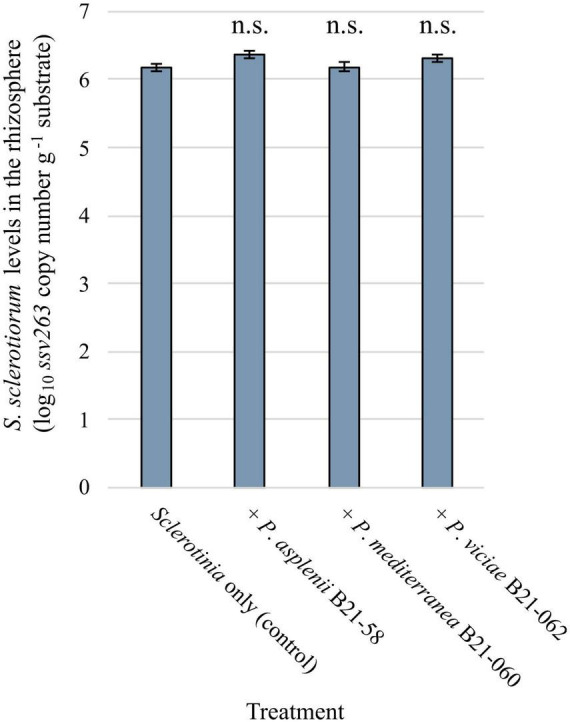
*Sclerotinia sclerotiorum* population levels in the rhizosphere of pot-grown lettuce inoculated with three inhibitory *Pseudomonas* strains estimated by quantitative PCR using the SSBZ primers and probe. Bars indicate the standard error of the mean; n.s., no statistically significant differences were found between a bacterial treatment and the control.

*Sclerotinia sclerotiorum* population levels in the rhizosphere of lettuce grown in pots and inoculated with antagonistic *Pseudomonas* strains and the pathogen were assessed by qPCR. A pair of primers and a TaqMan probe were used to target a fragment of the *ssv263* gene, which is specific to *S. sclerotiorum* (Figure 6). No significant differences in *ssv263* copy numbers were found between rhizosphere samples treated with the pathogen and *Pseudomonas* strains and rhizosphere samples containing the pathogen only. The average *ssv263* copy number was 1.83 × 10^6^ g^–1^ rhizosphere.

## 4 Discussion

In this study, nine *Pseudomonas* strains were identified for their ability to inhibit *S. sclerotiorum*’s growth under *in vitro* conditions and three were also able to reduce disease symptoms *in planta*. Their genomes were sequenced, allowing for an identification at the species level, and potential genetic determinants involved in their biocontrol capabilities were investigated.

These strains belong to the *P. corrugata*, *P. asplenii*, *P. mandelii*, and *P. protegens* phylogenomic subgroups, all belonging to the *P. fluorescens* group (Girard et al., 2021) known to contain most *Pseudomonas* spp. strains of biocontrol interest discovered so far (Oni et al., 2022). The *P. corrugata* subgroup was the most represented one, containing six of the nine identified antagonists. To our knowledge, only one *Pseudomonas* strain in this subgroup was previously shown to inhibit *S. sclerotiorum*: *Pseudomonas* sp. DF41 (Berry et al., 2010; Loewen et al., 2014). This subgroup is known to contain both plant-beneficial and plant-pathogenic strains (Catara, 2007; Gislason and de Kievit, 2020; Höfte, 2021). The *P. corrugata* species is known to contain opportunistic pathogens causing tomato pit necrosis, which have also been shown to display antimicrobial activity and biocontrol potential (Catara, 2007; Trantas et al., 2015). The *P. asplenii* subgroup was represented by a single antagonistic strain in our study, belonging to the *P. asplenii* species. This subgroup has not been extensively studied to date, but similarly to the *P. corrugata* one, it is known to contain plant pathogens (Höfte and Vos, 2006). The *P. mandelii* subgroup has been better characterized than the *P. asplenii* one and contains multiple strains of biocontrol interest (Höfte, 2021). Only one of these strains, *Pseudomonas arsenicoxydans* F9-7, has been previously shown effective against *S. sclerotiorum* (Meyer, 2022). Finally, the *P. protegens* subgroup is well known for containing biocontrol strains (Höfte, 2021), especially *P. protegens* Pf-5, which inhibits *S. sclerotiorum* (Balthazar et al., 2022). Since most of the identified antagonistic strains in our study belong to phylogenomic subgroups known to contain some plant pathogens, they could be pathogenic to lettuce. However, our results indicate that none of the nine antagonistic strains could cause visible symptoms on lettuce, suggesting that they could be safely used as biocontrol agents on this plant.

From these nine *Pseudomonas* strains, only three were able to significantly reduce *S. sclerotiorum* symptoms development under *in planta* conditions, both on detached leaves and in pot-grown plants. These strains are *Pseudomonas mediterranea* B21-060, *Pseudomonas viciae* B21-062 (both from the *P. corrugata* subgroup), and *P. asplenii* B21-058 (from the *P. asplenii* subgroup). Their biocontrol ability in the detached leave assay points to a direct inhibition mechanism of *S. sclerotiorum*, probably antibiosis, rather than an indirect one, such as the induction of systemic resistance in the plant. To better understand the mechanism at play, population levels of *S. sclerotiorum* in the rhizosphere of pot-grown lettuce inoculated with the three *Pseudomonas* strains were assessed by qPCR. Albeit disease symptoms were reduced in *Pseudomonas*-treated plants, *S. sclerotiorum* population levels were similar in those treatments and in the control inoculated with *S. sclerotiorum* only. While it is possible that DNA from dead *Sclerotinia* hyphae was amplified in the qPCR reactions, several studies show that extracellular DNA is rapidly degraded in soils (Herdina et al., 2004; Kunadiya et al., 2021). Therefore, the amount of DNA from dead *Sclerotinia* hyphae detected by the qPCR assays used in this study was most probably negligible. The results obtained suggest that biocontrol by the three *Pseudomonas* strains is not mediated by a direct reduction in the pathogen’s population. It rather points to a reduction in the ability of the pathogen to cause the disease, potentially impacting its virulence. Such a mechanism has been previously demonstrated in other *Pseudomonas* biocontrol agents effective against plant pathogens such as *Streptomyces scabiei* and *Pythium* spp. (de Souza et al., 2003; Arseneault et al., 2015; Arseneault and Filion, 2017). In these interactions, the implication of antimicrobial molecules in biocontrol was first demonstrated *in vitro*, similar to here. However, their *in planta* activity involved antimicrobial molecule concentrations that were most likely at subinhibitory levels, leading to reduced pathogen’s fitness and the alteration of their capacity to cause symptoms, without significant altering the pathogen population. In our study, in addition to antibiosis, we cannot completely rule out that induced systemic resistance may also be involved in the biocontrol abilities of the three strains, at least in the pot assay. Indeed, multiple *Pseudomonas* spp. have been shown to induce systemic resistance in various plant species against several pathogens (Pršić and Ongena, 2020).

To better understand the mechanism(s) leading to biocontrol under *in planta* conditions in our study, we explored the diversity of secondary metabolites potentially produced by these antagonistic *Pseudomonas* strains by identifying genes and BGCs involved in antibiotic biosynthesis in their genomes that are mostly absent from the genomes of 35 other *Pseudomonas* strains, non-antagonistic against *S. sclerotiorum* (Zboralski et al., 2022; listed in Supplementary Table 1).

The BGCs involved in the production of mycins and peptins, two categories of lipopeptides, were found in the genomes of almost all antagonistic strains and only in one strain unable to inhibit *S. sclerotiorum in vitro*. Lipopeptides are well known for their antimicrobial properties against a wide range of plant pathogens, including *S. sclerotiorum* and closely related fungi (Van Der Voort et al., 2015; Oni et al., 2022). They induce membrane leakage and/or an influx of ions in the affected cells by integrating into the plasma membrane and causing its rearrangement (Dimkić et al., 2022). Their activity can result in cell death or the disruption of cell division. Lipopeptides also contribute to bacterial colonization, protection against predators, biofilm formation, the induction of systemic resistance in plants, and sometimes, to virulence (Raaijmakers et al., 2010). The distinction between phytobeneficial and phytopathogenic lipopeptide producers is not strict and can depend on the type of lipopeptides produced (Girard et al., 2020). Lipopeptides that have been described as virulence factors in well-known *Pseudomonas* pathogens do not necessarily play the same role in non-pathogenic ones (Girard et al., 2020). The mycins and peptins potentially produced by the antagonistic strains under study were predicted *in silico* and included nunamycin, brasmycin, thanamycin, syringotoxin, nunapeptin, braspeptin, corpeptin, and fuscopeptin (Table 2). Some of these lipopeptides have been shown to display antifungal activity. For example, *P. viciae* 11K1 produces braspeptin, which inhibits *Botryosphaeria dothidea* (Zhao et al., 2019). Syringotoxin and fuscopeptin, both produced by *Pseudomonas fuscovaginae* UPB 264, inhibit *Botrytis cinerea* and *Rhodotorula pilimanae* (Ballio et al., 1996). *Pseudomonas* sp. SH-C52 produces thanamycin, which displays antagonistic activity against *Rhizoctonia solani* on sugarbeets and *Sclerotium rolfsii* on groundnuts (Le et al., 2012; Van Der Voort et al., 2015). Nunamycin is produced by *P. fluorescens* In5 and has been shown to inhibit the mycelial growth of *R. solani* (Michelsen et al., 2015). To date, corpeptin has been shown to be primarily a phytotoxic compound (Emanuele and Giorgio, 1998; Strano et al., 2015). It displays antimicrobial activity against *Bacillus megaterium*, but no antifungal activity has been associated yet with this molecule (Catara, 2007). However, corpeptin is structurally similar to sclerosin, a lipopeptide produced by *Pseudomonas* sp. DF41 that inhibits ascospore and sclerotia germination in co-culture with *S. sclerotiorum* (Berry et al., 2012). This lipopeptide may therefore display fungal inhibition capabilities.

Interestingly, seven out of nine antagonistic *Pseudomonas* strains identified in this study possess the BGC involved in brabantamide production, which is absent from 34 out of the 35 *Pseudomonas* strains unable to inhibit *S. sclerotiorum* (Figure 3). Brabantamides are glycosylated lipopeptides with phospholipase inhibitory activity showing great antimicrobial potential (Schmidt et al., 2014). The main compound of this 3-member family is brabantamide A (Schmidt et al., 2014). This antibiotic is known for its inhibition capabilities against several pathogenic Gram-positive bacteria such as *Staphylococcus aureus* and *Arthrobacter crystallopoietes* (Van Der Voort et al., 2015). Studies on its antifungal activity have been limited to the phytopathogenic fungus *Microdochium nivale*, where it was shown to reduce its infection (Andersson et al., 2012).

*Pseudomonas* sp. B21-059 (*P. protegens* subgroup) was the only one of the nine antagonistic strains to lack BGCs involved in the production of peptins and mycins. This strain also displayed a slightly different BGC profile than strains belonging to the same subgroup that were unable to inhibit *S. sclerotiorum*. It carries the rhizoxin cluster. Rhizoxin analogs are macrolides notably displaying inhibition abilities against plant pathogens, for instance against the ascomycete *Fusarium oxysporum* (Takeuchi et al., 2015). The rhizoxin analog(s) potentially produced by B21-059 may act synergistically with other antibiotics against *S. sclerotiorum*, such as 2,4-DAPG and pyrrolnitrin, whose BGCs are also found in the strain’s genome. In another strain belonging to the *P. protegens* subgroup, *P. protegens* Pf-5, such a synergistic effect between 2,4-DAPG, pyrrolnitrin, and rhizoxins was demonstrated against *Fusarium* isolates (Quecine et al., 2016).

Six out of the nine antagonistic *Pseudomonas* strains did not significantly reduce *S. sclerotiorum* lesion diameters on detached lettuce leaves, including the strain that showed the highest inhibition zone in confrontation assays against the pathogen. Since biocontrol agents need to display ecological competence to ensure their antagonistic activity in their target habitat, it is possible that these six strains, which were all isolated from soils, were not able to settle in the phyllosphere. Alternatively, they may have colonized the phyllosphere but failed to produce antifungal compounds. Antibiotic production indeed depends on specific signals and external chemical cues such as nutritional and environmental factors (Scherlach and Hertweck, 2009). These factors influence global regulatory systems, such as the Gac-Rsm pathway (Ferreiro and Gallegos, 2021), LuxR-type regulators (Girard et al., 2020), and quorum sensing systems (Venturi, 2006) which in turn control the expression of genes involved in the production of antimicrobial compounds in many *Pseudomonas* spp. For example, Humair et al. (2009) found that the expression of genes involved in the production of antimicrobial compounds such as HCN in *P. fluorescens* CHA0 was significantly lower at high temperature. The phyllosphere is known for being more exposed to specific abiotic stresses, such as high temperature, UV radiation, and oxidative stress from photosynthesis, compared to the rhizosphere (Zboralski et al., 2023). Our study was not specifically designed to determine the role of these different factors in the biocontrol activity of the *Pseudomonas* strains on detached leaves. This will require further examination to better understand why only some strains displaying strong antagonistic activity *in vitro* against *S. sclerotiorum* significantly inhibited the pathogen on detached leaves while others did not.

## 5 Conclusion

Nine *Pseudomonas* strains able to inhibit *S. sclerotiorum* under *in vitro* conditions were identified in this study, including three that demonstrated great biocontrol potential in lettuce against the pathogen. Their genomes were sequenced, enabling a reliable species-level identification, and doubling the number of genomes publicly available for *Pseudomonas* strains known for their inhibitory abilities against *S. sclerotiorum*. Biocontrol-related traits potentially involved in the inhibition were identified in the nine genomes. The presence of BGCs involved in the biosynthesis of mycin and peptin lipopeptides, as well as the antibiotic brabantamide, strongly correlated with the antagonistic activity observed. The use of reverse genetics, analytical chemistry, and more complex plant system experiments will however be needed to improve our understanding of the biocontrol capabilities of these strains and progress toward the development of *Pseudomonas* inoculants effective against *S*. *sclerotiorum* in lettuce and other crops.

## Data availability statement

The datasets presented in this study can be found under the accession number PRJNA779433 (BioProject) in the following online repository: https://www.ncbi.nlm.nih.gov/genbank/.

## Author contributions

DA: Conceptualization, Formal analysis, Investigation, Methodology, Writing – original draft, Writing – review & editing. AZ: Conceptualization, Formal analysis, Investigation, Methodology, Writing – original draft, Writing – review & editing. MCi: Conceptualization, Methodology, Writing – review & editing. MCa: Conceptualization, Methodology, Writing – review & editing. AB: Conceptualization, Methodology, Writing – review & editing. JB: Software, Writing – review & editing. CB: Conceptualization, Writing – review & editing. MF: Conceptualization, Funding acquisition, Methodology, Supervision, Writing – original draft, Writing – review & editing.
